# Personality Traits and Illness Acceptance in Patients with Cancer and Cardiovascular Disease: A Cross-Sectional Study from Poland

**DOI:** 10.3390/jcm15197435

**Published:** 2026-09-24

**Authors:** Robert Jan Łuczyk, Marta Łuczyk, Dorota Weber, Kamil Sikora, Anna Charuta

**Affiliations:** 1 Institute of Health Sciences, Faculty of Medical and Health Sciences, University of Siedlce, 08-110 Siedlce, Poland; anna.charuta@uws.edu.pl; 2 Long-Term Care Nursing Department, Chair of Preventive Nursing, Faculty of Health Sciences, Medical University of Lublin, Ul. Chodźki 7, 20-093 Lublin, Poland; marta.luczyk@umlub.edu.pl; 3 Faculty of Health Sciences, Lublin University of Technology, WSEI in Lublin, Projektowa 4, 20-209 Lublin, Poland; dorota.weber@wsei.pl; 4 Department of Internal Medicine and Internal Nursing, Chair of Preventive Nursing, Faculty of Health Sciences, Medical University of Lublin, Ul. Chodźki 7, 20-093 Lublin, Poland; kamil.sikora@umlub.edu.pl

**Keywords:** illness acceptance, personality traits, NEO-FFI, Acceptance of Illness Scale, cancer, cardiovascular disease, moderated regression, psycho-oncology

## Abstract

**Background:** Illness acceptance is a key psychological factor shaping adjustment to chronic disease, and personality traits are among its established correlates. Whether the diagnostic context—oncological versus cardiovascular disease—moderates the relationship between personality and illness acceptance remains insufficiently studied. This study examined associations between the Big Five personality traits (NEO-FFI) and illness acceptance (AIS) in patients with cancer and cardiovascular disease, and tested whether diagnosis moderates these associations. **Methods:** A cross-sectional study was conducted between January 2024 and May 2026 at the Oncology Centre of the Lublin Region, University Hospital No. 4, Lublin, and University Hospital No. 1, Lublin. The sample comprised 631 patients: 316 with a cancer diagnosis and 315 with cardiovascular disease. Personality traits were measured with the NEO Five-Factor Inventory (NEO-FFI) and illness acceptance with the Acceptance of Illness Scale (AIS). An unadjusted moderated multiple regression was used, with personality traits as predictors, diagnosis (0 = cancer, 1 = cardiovascular disease) as the moderator, and illness acceptance as the outcome; age, sex, and illness duration were not included as covariates, and all continuous variables were standardized. Analyses were performed in R (version 4.2.2) via RStudio (version 2022.12.0), with α = 0.05. **Results:** Patients with cardiovascular disease scored higher on neuroticism and lower on openness, agreeableness, and conscientiousness than patients with cancer (all *p* < 0.05); groups did not differ in extraversion or overall illness acceptance. The unadjusted moderated regression model was significant, F (11, 619) = 9.88, *p* < 0.001, explaining 14.9% of the variance in illness acceptance (adjusted R^2^ = 0.134). The diagnosis coefficient was positive and statistically significant in the unadjusted model (β = 0.23, *p* = 0.002), while neuroticism was negatively associated with illness acceptance (β = −0.29, *p* < 0.001). A significant openness × diagnosis interaction was observed (β = −0.22, *p* = 0.008); the model-estimated conditional coefficients for openness were β = 0.09 in the cancer group and β = −0.13 in the cardiovascular disease group. Formal inferential tests of the within-group slopes were not performed, and the model was not adjusted for age, sex, or illness duration. **Conclusions:** In this cross-sectional study, neuroticism was negatively associated with illness acceptance in the unadjusted model, whereas the openness × diagnosis interaction was exploratory. The conditional coefficients pointed in opposite directions across diagnostic groups, but the within-group slopes were not formally tested and the model was not adjusted for age, sex, or illness duration. These findings are hypothesis-generating and require confirmation in adjusted and longitudinal studies before informing diagnosis-specific clinical support.

## 1. Introduction

Acceptance of illness is a central construct in health psychology, describing the degree to which a person adapts to the limitations, losses, and altered self-image imposed by a chronic condition [1]. First operationalized by Felton, Revenson, and Hinrichsen as part of a broader model of stress and coping among chronically ill adults, illness acceptance captures a person’s ability to accommodate disease-related restrictions without a pervasive loss of self-worth [1]. A higher level of acceptance has repeatedly been linked to better psychological adjustment, greater treatment adherence, and improved quality of life across a wide range of chronic conditions, from cancer to cardiovascular and renal disease [2,3,4,5,6].

Personality traits, conceptualized within the Five-Factor Model, are among the most extensively studied dispositional correlates of how patients respond to chronic illness [7,8]. Neuroticism—the tendency toward negative affect, anxiety, and emotional instability—has been consistently associated with poorer psychological adjustment, greater symptom burden, and lower quality of life in both oncological and cardiological populations [9,10,11,12]. Conversely, conscientiousness and agreeableness have generally been linked to more adaptive health behaviours and better long-term outcomes [11,13,14,15], while findings for openness and extraversion have been more mixed and context-dependent [9,15,16].

Cancer and cardiovascular disease represent two of the most prevalent and burdensome chronic illness contexts, yet they differ substantially in symptom trajectory, treatment demands, and the cultural meaning attached to the diagnosis. Cancer is frequently experienced as an acute existential threat with a fluctuating but often intensive treatment course, whereas cardiovascular disease more often follows a protracted, fluctuating course requiring sustained lifestyle modification [11,12,17,18,19]. Prior work has documented associations between illness acceptance and personality separately within oncological [2,9,10,16,20] and cardiovascular [3,4,5,6,11,12,13,17,18,19] populations, and between personality and quality of life in other chronic conditions such as chronic kidney disease [15,21] and diabetes-adjacent disease groups [22,23]. However, direct comparisons of how the relationship between personality and illness acceptance differs by diagnostic context—that is, whether diagnosis moderates this relationship—remain scarce.

This gap is clinically relevant: if the personality correlates of illness acceptance differ between cancer and cardiovascular disease, psychological support and screening protocols built on a single, disease-general profile may be poorly calibrated for one or both populations. Nurses and other clinicians are often best positioned to detect early signs of poor psychological adjustment [24,25,26], and a clearer picture of which personality traits carry risk in which diagnostic context could sharpen these screening efforts.

The present study had two objectives: (1) to compare the Big Five personality traits and illness acceptance between patients with cancer and those with cardiovascular disease; (2) to examine whether the diagnostic group moderated associations between personality traits and illness acceptance. Based on the established transdiagnostic role of negative affectivity, we expected neuroticism to be inversely associated with illness acceptance. Because prior evidence did not support clear disease-specific predictions for all five traits, the interaction analyses were treated as exploratory.

## 2. Materials and Methods

### 2.1. Study Design and Participants

This multicentre cross-sectional study was conducted between January 2024 and May 2026 at three clinical sites in Lublin, Poland: the Oncology Centre of the Lublin Region, University Hospital No. 4 in Lublin, and University Hospital No. 1 in Lublin. The analytic sample comprised 631 adult patients, including 316 (50.1%) with a confirmed cancer diagnosis and 315 (49.9%) with a confirmed cardiovascular disease diagnosis. Participants formed a non-probability clinical sample recruited in the participating centres. Eligibility for the present analysis required age ≥ 18 years, assignment to one of the two prespecified diagnostic groups on the basis of the medical record, capacity to complete self-report questionnaires, informed consent, and sufficient NEO-FFI and AIS data for scoring. Records lacking a confirmed group diagnosis or sufficient questionnaire data were not included. The available dataset classified diagnoses at the group level (cancer or cardiovascular disease); tumour site, cancer stage, individual cardiovascular diagnosis, and treatment phase were not consistently available and were therefore not used for subgroup analyses.

### 2.2. Measures

#### 2.2.1. NEO Five-Factor Inventory (NEO-FFI)

Personality traits were assessed with the Polish adaptation of the NEO Five-Factor Inventory (NEO-FFI), a 60-item self-report measure of neuroticism, extraversion, openness to experience, agreeableness, and conscientiousness [7,8,27]. Each domain comprises 12 items rated on a 5-point Likert scale. Raw domain scores were used in descriptive and group-comparison analyses and were standardized before regression modelling. Internal consistency in the present sample ranged from acceptable to excellent (Cronbach’s α = 0.668–0.907; Table 1).

#### 2.2.2. Acceptance of Illness Scale (AIS)

Illness acceptance was measured using the Polish adaptation of the Acceptance of Illness Scale (AIS), developed by Felton, Revenson, and Hinrichsen and adapted for Polish use by Juczyński [1,28]. The eight items address limitations, loss of self-sufficiency, dependence on others, and diminished self-esteem associated with poor health. Items are rated on a 5-point scale, yielding a total score from 8 to 40; higher scores indicate greater illness acceptance and better psychological adaptation. Internal consistency in the present sample was good (Cronbach’s α = 0.817).

### 2.3. Statistical Analysis

Descriptive statistics (means, standard deviations, skewness, and kurtosis) were computed for all NEO-FFI subscales and for illness acceptance. Group differences between cancer and cardiovascular disease patients on categorical demographic variables were assessed with the chi-squared test (Cramér’s V as effect size); differences on ordinal/age variables were assessed with the Mann–Whitney U test (η^2^ as effect size); differences in personality traits and illness acceptance were assessed with independent-samples *t*-tests (Cohen’s d as effect size).

To test the central hypothesis that diagnosis moderates the relationship between personality and illness acceptance, an unadjusted moderated multiple regression model was estimated with illness acceptance as the dependent variable, the five personality traits as predictors, diagnosis (0 = cancer, 1 = cardiovascular disease) as the moderator, and the five trait × diagnosis interaction terms. All continuous predictors were standardized (z-scored) prior to analysis; the moderator was dummy-coded [29,30]. No demographic or clinical covariates were included; in particular, age, sex, and illness duration were not entered into the model. Multicollinearity was evaluated using the generalized variance inflation factor (GVIF), appropriate in the presence of categorical predictors and interaction terms [31]; the highest adjusted GVIF was 1.183 (conscientiousness), indicating no problematic collinearity. Residual diagnostics—homoscedasticity (residuals-vs-fitted plot), normality of residuals (Q–Q plot), and influential-observation screening (Cook’s distance)—were inspected visually (Figure 1); the maximum Cook’s distance was 0.038, indicating no unduly influential observations. The significant openness × diagnosis interaction was visualized using model-estimated conditional coefficients for openness at each level of diagnosis [30]; formal inferential simple-slope tests were not performed. All analyses were conducted in R (version 4.2.2) via RStudio (version 2022.12.0), with statistical significance set a priori at α = 0.05.

## 3. Results

### 3.1. Sample Characteristics

The study group comprised 631 patients: 316 (50.1%) with cancer and 315 (49.9%) with cardiovascular disease (Table 2). Women constituted 70.3% of the cancer group and 53.0% of the cardiovascular group; the sex distribution differed significantly (χ^2^ (1) = 19.82, *p* < 0.001), with a small association (Cramér’s V = 0.177). Age distributions differed slightly (U = 45,263.50, *p* = 0.043, η^2^ = 0.006), whereas education and place of residence did not differ between groups (both *p* > 0.10). Illness duration differed substantially: the cardiovascular group had a higher mean rank (401.42) than the cancer group (230.85; U = 22,862.00, *p* < 0.001, η^2^ = 0.238). These between-group differences were considered as limitations when interpreting the unadjusted moderation model; age, sex, and illness duration were not included as covariates.

### 3.2. Descriptive Statistics and Correlations Among Study Variables

Descriptive statistics and Pearson correlations for the five NEO-FFI subscales and illness acceptance across the whole sample are presented in Table 1. Internal consistency was acceptable to excellent for all subscales, ranging from α = 0.668 (agreeableness) to α = 0.907 (neuroticism). Illness acceptance correlated negatively with neuroticism (r = −0.343) and positively with extraversion (r = 0.239), openness (r = 0.088), agreeableness (r = 0.181), and conscientiousness (r = 0.194). All correlations were statistically significant at *p* < 0.001, with the exception of the correlation between openness and illness acceptance (*p* = 0.027).

### 3.3. Between-Group Differences in Personality Traits and Illness Acceptance

Patients with cancer and patients with cardiovascular disease differed significantly, though modestly, on four of the five personality traits (Table 3). Cardiovascular patients scored higher on neuroticism (t (629) = 3.467, *p* = 0.001, d = 0.276) and lower on openness (t (629) = 2.677, *p* = 0.008, d = 0.213), agreeableness (t (629) = 3.954, *p* < 0.001, d = 0.315), and conscientiousness (t (629) = 2.412, *p* = 0.016, d = 0.192) than cancer patients. Groups did not differ significantly in extraversion (t (617) = 1.809, *p* = 0.071, d = 0.144) or in overall illness acceptance (t (602) = 1.560, *p* = 0.119, d = 0.124).

### 3.4. Moderated Regression Analysis

A moderated multiple regression model was estimated with illness acceptance as the outcome, the five NEO-FFI traits and diagnosis as predictors, and the five trait × diagnosis interaction terms (Table 4). The generalized variance inflation factor did not indicate problematic multicollinearity among predictors, including the categorical diagnosis variable; the highest adjusted GVIF was observed for conscientiousness (1.183). Visual inspection of residual diagnostics supported the model’s assumptions: residuals were homoscedastically distributed across fitted values, the Q–Q plot indicated an approximately normal distribution of residuals with only minor deviation at the extremes, and the maximum Cook’s distance (0.038) indicated no unduly influential observations (Figure 1).

The overall model was statistically significant, F (11, 619) = 9.88, *p* < 0.001, although it explained a modest proportion of variance in illness acceptance (R^2^ = 0.149; adjusted R^2^ = 0.134). The diagnosis coefficient was positive and statistically significant in the unadjusted model (β = 0.23, SE = 0.08, *p* = 0.002), indicating higher model-predicted illness-acceptance scores in the cardiovascular disease group at the reference values of the standardized personality predictors. Because age, sex, and illness duration were not included as covariates, this coefficient should not be interpreted as an independent association. Neuroticism was the strongest predictor in the model (β = −0.29, SE = 0.07, *p* < 0.001), corresponding to a 0.29-SD lower model-predicted illness-acceptance score per 1-SD increase in neuroticism in the cancer reference group. No statistically significant evidence of a diagnosis difference in this slope was observed (neuroticism × diagnosis: *p* = 0.675). None of extraversion, openness, agreeableness, or conscientiousness reached significance as main effects (all *p* > 0.10). Of the five interaction terms, only openness × diagnosis was statistically significant (β = −0.22, SE = 0.08, t = −2.66, *p* = 0.008, 95% CI [−0.38, −0.06]). With cancer coded as the reference group, the model-estimated conditional coefficient for openness was β = 0.09 in the cancer group and β = −0.13 in the cardiovascular disease group (0.09–0.22; Figure 2). These conditional coefficients indicate opposite fitted directions, but they are not formal simple-slope tests; separate coefficients, standard errors, confidence intervals, and *p*-values for the two within-group slopes were not calculated in this revision. The remaining trait × diagnosis terms were not statistically significant (all *p* > 0.40). The interaction was estimated in an unadjusted model and was not examined after adjustment for age, sex, or illness duration; therefore, it should be considered exploratory and hypothesis-generating.

## 4. Discussion

This study compared personality traits and illness acceptance between patients with cancer and patients with cardiovascular disease and examined whether the diagnostic group moderated the associations between personality and illness acceptance. Three findings stand out. First, cardiovascular patients showed a modestly different personality profile than cancer patients—higher neuroticism and lower openness, agreeableness, and conscientiousness—despite reporting numerically similar overall illness acceptance. Second, neuroticism was the strongest negative predictor in the unadjusted model. Third, the unadjusted model yielded a statistically significant openness × diagnosis interaction, with model-estimated conditional coefficients pointing in opposite directions in the two diagnostic groups. Because formal simple-slope tests were not performed and age, sex, and illness duration were not included as covariates, the interaction should be interpreted as exploratory rather than as evidence of established diagnosis-specific effects The higher neuroticism observed among cardiovascular patients is consistent with prior evidence linking neuroticism to cardiovascular risk, symptom burden, and poorer prognosis [11,12,17,19]. Combined with the markedly longer illness duration in this group, this pattern may reflect either a pre-existing vulnerability that elevates cardiovascular risk, or an accumulating psychological burden of living with a long-term, fluctuating condition—a distinction this cross-sectional design cannot resolve. Similarly, the lower conscientiousness and agreeableness observed among cardiovascular patients align with evidence that low conscientiousness is a broad upstream risk factor for cardiovascular morbidity and mortality [11,13,19], though whether this reflects cause, consequence, or shared confounding remains an open question that would benefit from longitudinal replication.

In the present study, the negative association between neuroticism and illness acceptance was observed in the unadjusted model and is consistent with findings from cancer populations [9,10], cardiovascular populations [11,12,17], and other chronic illness groups such as chronic kidney disease [15,21] and general elderly chronic-disease samples [22]. This convergence is compatible with the possibility that neuroticism represents a transdiagnostic vulnerability factor for poorer adjustment to chronic illness. However, because the present model was not adjusted for age, sex, or illness duration, and because the study was cross-sectional, this interpretation remains provisional rather than definitive. The openness × diagnosis interaction is, to our knowledge, a less frequently reported finding and merits cautious interpretation. In the unadjusted model, the interaction term was statistically significant (β = −0.22, SE = 0.08, *p* = 0.008, 95% CI [−0.38, −0.06]), and the model-estimated conditional coefficients pointed in opposite directions in the cancer group (β = 0.09) and the cardiovascular disease group (β = −0.13). However, formal inferential tests of the two within-group slopes were not performed, and the model was not adjusted for age, sex, or illness duration. The observed pattern is therefore hypothesis-generating and cannot establish that openness is beneficial in cancer or detrimental in cardiovascular disease. The proposed explanation—that cognitive flexibility may be more relevant to information-dense cancer treatment, whereas cardiovascular adjustment may depend more on sustained behavioural routines—remains speculative [16,32]. Future adjusted and longitudinal studies should examine these possible mechanisms directly. Clinically, the present findings should not be used to guide individual treatment decisions. The negative association with neuroticism may justify further investigation of psychological vulnerability in both groups, but it does not establish a diagnosis-independent clinical risk marker. Likewise, the openness × diagnosis pattern should not yet be used to recommend disease-specific communication or behavioural-support strategies because it was derived from an unadjusted model and was not supported by formal simple-slope tests. Nurses and other clinicians remain well positioned to recognize poor adjustment and refer patients for comprehensive psychological assessment [24,25,26].

### Strengths and Limitations

Strengths of this study include the relatively large clinical sample, the direct comparison of two clinically important diagnostic groups, the use of established Polish adaptations of psychometric instruments, and the application of a moderated regression framework accompanied by collinearity and residual diagnostics.

Several limitations should be considered. First, the cross-sectional design precludes causal or temporal inference. Second, participants were recruited using a non-probability sampling approach from clinical centres in a single geographic region, which limits generalisability. Third, the dataset classified participants only into broad cancer and cardiovascular disease groups; tumour site and stage, specific cardiovascular diagnosis, disease severity, treatment phase, and comorbidity burden were not consistently available. Residual clinical heterogeneity may therefore have influenced the observed associations.

Fourth, illness duration differed substantially between the groups (U = 22,862.00, *p* < 0.001, η^2^ = 0.238). The groups also differed in sex distribution (χ^2^ (1) = 19.82, *p* < 0.001, Cramér’s V = 0.177) and age distribution (U = 45,263.50, *p* = 0.043, η^2^ = 0.006). Age, sex, and illness duration were not included as covariates; therefore, the diagnosis coefficient and the openness × diagnosis interaction should be interpreted as unadjusted associations that may be affected by residual confounding.

Fifth, formal inferential tests of the conditional within-group slopes for the openness × diagnosis interaction were not performed. The reported values of β = 0.09 for the cancer group and β = −0.13 for the cardiovascular disease group should therefore be understood as model-estimated conditional coefficients, rather than as separately confirmed group-specific effects.

Finally, all main measures were self-reported and may be subject to response bias and common-method bias. These limitations mean that the moderation finding should be considered exploratory and hypothesis-generating and should be tested in adjusted, longitudinal, and independently replicated studies.

## 5. Conclusions

In this cross-sectional study, neuroticism was negatively associated with illness acceptance in the unadjusted model, whereas an openness × diagnosis interaction was observed. The model-estimated conditional coefficients for openness pointed in opposite directions in the cancer and cardiovascular disease groups, but within-group simple slopes were not formally tested and the moderation model was not adjusted for age, sex, or illness duration. Accordingly, these findings are exploratory and hypothesis-generating; they require confirmation in adjusted, longitudinal, and independently replicated studies before informing diagnosis-specific clinical practice.

## Figures and Tables

**Figure 1:**
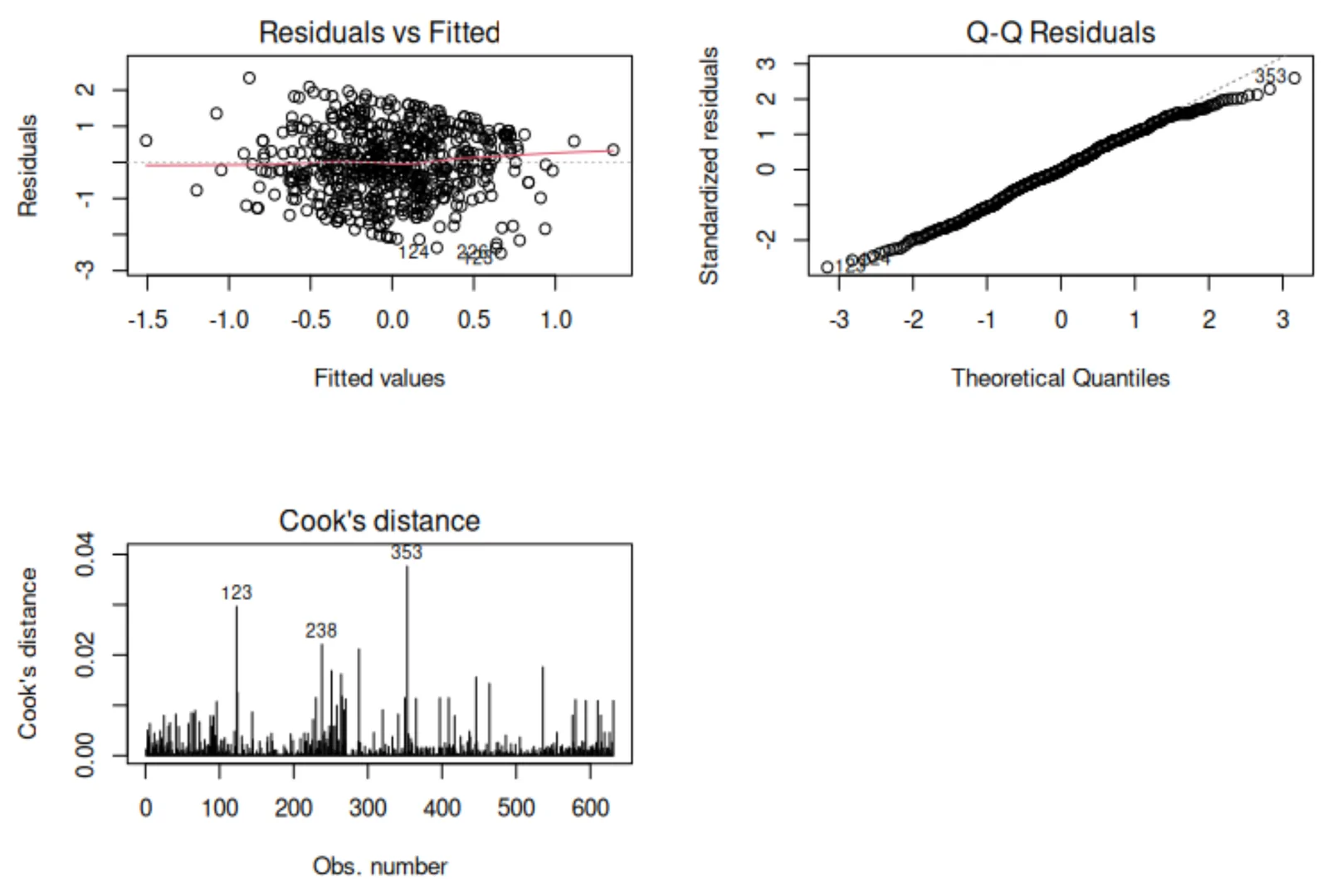
Regression diagnostics for the moderated regression model: residuals vs. fitted values (top left), Q–Q plot of standardized residuals (top right), and Cook’s distance by observation number (bottom).

**Figure 2:**
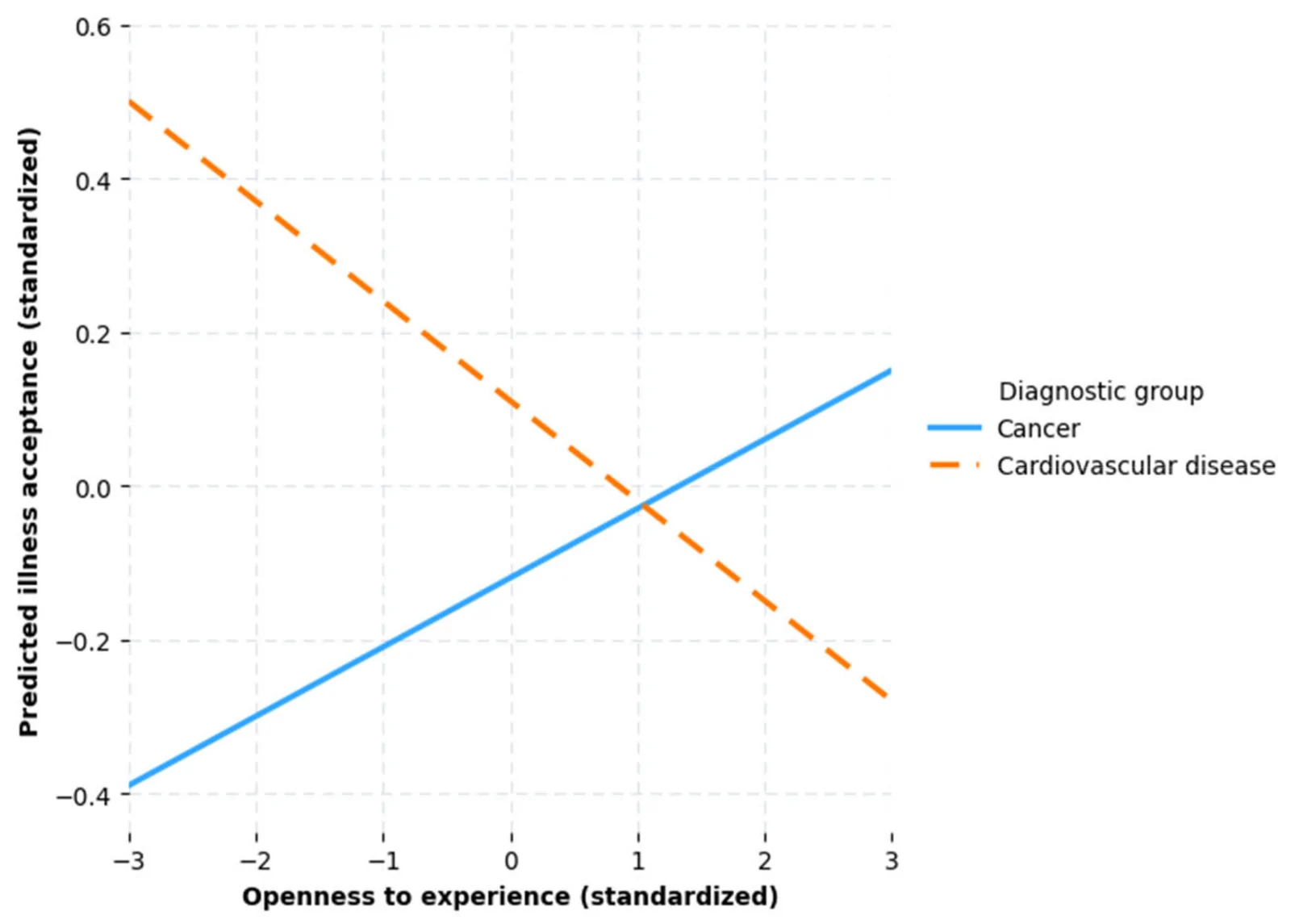
Interaction between openness to experience and diagnosis in predicting illness acceptance. Lines represent the model-estimated (not separately significance-tested) slope of openness within each diagnostic group.

**Table 1:** Descriptive statistics and Pearson correlations among NEO-FFI subscales and illness acceptance (N = 631).

Variable	M	SD	Skew	Kurt	Cronbach’s α	2	3	4	5	6
1. Neuroticism	23.75	7.50	0.018	−0.139	0.907	−0.510	−0.266	−0.405	−0.428	−0.343
2. Extraversion	25.54	7.18	−0.251	0.041	0.778	1	0.374	0.379	0.444	0.239
3. Openness	23.42	6.38	−0.128	0.116	0.758		1	0.229	0.172	0.088
4. Agreeableness	29.62	6.90	−0.625	0.228	0.668			1	0.531	0.181
5. Conscientiousness	31.62	7.20	−0.473	0.245	0.787				1	0.194
6. Illness acceptance	25.64	8.44	−0.155	−0.859	0.817					1

**Table 2:** Sociodemographic characteristics of patients with cancer and cardiovascular disease.

Variable	Category	Cancer (n = 316), n (%) Within Group	CVD (n = 315), n (%) Within Group	Total (N = 631), n (%)	Test Statistic
Sex	Female	222 (70.3%)	167 (53.0%)	389 (61.6%)	χ^2^ (1) = 19.82; *p* < 0.001; V = 0.177
	Male	94 (29.7%)	148 (47.0%)	242 (38.4%)	
Age	<31 years	18 (5.7%)	14 (4.4%)	32 (5.1%)	U = 45,263.50; *p* = 0.043; η^2^ = 0.006
	31–40 years	36 (11.4%)	38 (12.1%)	74 (11.7%)	
	41–50 years	81 (25.6%)	70 (22.2%)	151 (23.9%)	
	51–60 years	103 (32.6%)	85 (27.0%)	188 (29.8%)	
	61–70 years	66 (20.9%)	81 (25.7%)	147 (23.3%)	
	71–80 years	8 (2.5%)	19 (6.0%)	27 (4.3%)	
	>80 years	4 (1.3%)	8 (2.5%)	12 (1.9%)	
Education	Primary	23 (7.3%)	31 (9.8%)	54 (8.6%)	χ^2^ = 6.00; *p* = 0.112; V = 0.098
	Vocational	65 (20.6%)	85 (27.0%)	150 (23.8%)	
	Secondary	111 (35.1%)	101 (32.1%)	212 (33.6%)	
	Higher	117 (37.0%)	98 (31.1%)	215 (34.1%)	
Place of residence	Urban	210 (66.5%)	213 (67.6%)	423 (67.0%)	χ^2^ = 0.097; *p* = 0.756; V = 0.012
	Rural	106 (33.5%)	102 (32.4%)	208 (33.0%)	
Illness duration	<1 year	116 (36.7%)	25 (7.9%)	141 (22.3%)	U = 22,862.00; *p* < 0.001; η^2^ = 0.238
	1–3 years	147 (46.5%)	101 (32.1%)	248 (39.3%)	
	4–6 years	30 (9.5%)	72 (22.9%)	102 (16.2%)	
	7–10 years	17 (5.4%)	53 (16.8%)	70 (11.1%)	
	11–15 years	5 (1.6%)	33 (10.5%)	38 (6.0%)	
	>15 years	1 (0.3%)	31 (9.8%)	32 (5.1%)	

**Table 3:** Differences between cancer and cardiovascular disease patients in personality traits and illness acceptance.

Variable	Cancer		CVD		Student’s *t*-Test			
	M	SD	M	SD	t	df	*p*	d
Neuroticism	22.72	7.69	24.78	7.18	3.467	629	0.001	0.276
Extraversion	26.05	7.66	25.02	6.64	1.809	617	0.071	0.144
Openness	24.09	6.53	22.74	6.15	2.677	629	0.008	0.213
Agreeableness	30.69	6.88	28.54	6.76	3.954	629	<0.001	0.315
Conscientiousness	32.30	7.23	30.93	7.11	2.412	629	0.016	0.192
Illness acceptance	25.12	7.51	26.17	9.27	1.560	602	0.119	0.124

**Table 4:** Moderated linear regression predicting illness acceptance from personality traits, diagnosis, and their interactions.

Predictor	β	SE	t	*p*	95% CI
Constant	−0.12	0.05	−2.19	0.029	[−0.22, −0.01]
Diagnosis (cardiovascular) ^1^	0.23	0.08	3.05	0.002	[0.08, 0.38]
Neuroticism	−0.29	0.07	−4.14	<0.001	[−0.43, −0.15]
Extraversion	0.03	0.07	0.47	0.635	[−0.10, 0.16]
Openness	0.09	0.06	1.59	0.112	[−0.02, 0.21]
Agreeableness	0.01	0.07	0.11	0.909	[−0.13, 0.15]
Conscientiousness	0.02	0.07	0.21	0.836	[−0.13, 0.16]
Neuroticism × Diagnosis	−0.04	0.09	−0.42	0.675	[−0.22, 0.15]
Extraversion × Diagnosis	0.07	0.09	0.78	0.437	[−0.11, 0.26]
Openness × Diagnosis	−0.22	0.08	−2.66	0.008	[−0.38, −0.06]
Agreeableness × Diagnosis	0.04	0.09	0.44	0.664	[−0.14, 0.23]
Conscientiousness × Diagnosis	0.03	0.10	0.30	0.767	[−0.16, 0.22]

Note: Reference group: cancer; comparison group (coded ^1^): cardiovascular disease. Continuous predictors were standardized. All coefficients are from the unadjusted model; age, sex, and illness duration were not included as covariates. The model-estimated conditional coefficients for openness were not accompanied by formal simple-slope inferential tests. Model summary: R^2^ = 0.149; adjusted R^2^ = 0.134; F (11, 619) = 9.88; *p* < 0.001.

## Data Availability

The data supporting the findings of this study are available from the corresponding author upon reasonable request, subject to ethical and institutional restrictions protecting participant confidentiality.
